# Cardiology researchers’ practices and perceived barriers to open science: an international survey

**DOI:** 10.1136/openhrt-2023-002433

**Published:** 2024-01-17

**Authors:** Kelly D Cobey, Mohsen Alayche, Sara Saba, Nana Yaa Barnes, Sanam Ebrahimzadeh, Emilio Alarcón, Benjamin Hibbert, David Moher

**Affiliations:** 1 University of Ottawa Heart Institute, Ottawa, Ontario, Canada; 2 University of Ottawa, Ottawa, Ontario, Canada; 3 Department of Medicine, University of Ottawa, Ottawa, Ontario, Canada; 4 Health Sciences University of Ottawa, Ottawa, Ontario, Canada; 5 Ottawa Hospital Research Institute, Ottawa, Ontario, Canada; 6 Department of Biochemistry, Microbiology and Immunology, University of Ottawa, Ottawa, Ontario, Canada; 7 Department of Cardiovascular Medicine, Mayo Clinic, Rochester, Minnesota, USA

**Keywords:** Research Design, Translational Medical Research, Ethics, Medical, Education, Medical

## Abstract

**Objective:**

Open science is a movement and set of practices to conduct research more transparently. Implementing open science will significantly improve public access and supports equity. It also has the potential to foster innovation and reduce duplication through data and materials sharing. Here, we survey an international group of researchers publishing in cardiovascular journals regarding their perceptions and practices related to open science.

**Methods:**

We identified the top 100 ‘Cardiology and Cardiovascular Medicine’ subject category journals from the SCImago journal ranking platform. This is a publicly available portal that draws from Scopus. We then extracted the corresponding author’s name and email from all articles published in these journals between 1 March 2021 and 1 March 2022. Participants were sent a purpose-built survey about open science. The survey contained primarily multiple choice and scale-based questions for which we report count data and percentages. For the few text-based responses we conducted thematic content analysis.

**Results:**

198 participants responded to our survey. Participants had a mean response of 6.8 (N=197, SD=1.8) on a 9-point scale with endpoints, not at all familiar (1) and extremely familiar (9), when indicating how familiar they were with open science. When asked about where they obtained open science training, most participants indicated this was done on the job self-initiated while conducting research (n=103, 52%), or that they had no formal training with respect to open science (n=72, 36%). More than half of the participants indicated they would benefit from practical support from their institution on how to perform open science practices (N=106, 54%). A diversity of barriers to each of the open science practices presented to participants were acknowledged. Participants indicated that funding was the most essential incentive to adopt open science.

**Conclusions:**

It is clear that policy alone will not lead to the effective implementation of open science. This survey serves as a baseline for the cardiovascular research community’s open science performance and perception and can be used to inform future interventions and monitoring.

**WHAT IS ALREADY KNOWN ON THIS TOPIC**

- A series of open science mandates (eg, open access, open data) are being introduced rapidly by research funders to support research transparency.
- Cardiovascular research, like much of medical research, is typically conducted in a ‘closed’ fashion with many research outputs never being shared in any way, meaning that new mandates will require significant behaviour change.

**WHAT THIS STUDY ADDS**

- This study provides the first data specific to the international cardiovascular research community on knowledge of open science and barriers and facilitators to its implementation.

**HOW THIS STUDY MIGHT AFFECT RESEARCH, PRACTICE OR POLICY**

- Effective implementation of open science will require consorted discussion about diverse community stakeholders; the present research provides an important starting point by capturing the perspective of cardiovascular researchers on open science.

## Introduction

Open science is a movement to make the research lifecycle accessible to all—including practices such as open access publishing, data and code sharing and open (source) materials sharing. There is growing momentum globally to see open science practices more firmly embedded into the research ecosystem, with several jurisdictions having introduced policies and roadmaps to foster effective implementation.^1–5^ Previous research suggests that up to 85% of research conducted is wasted,^6^ and that the scientific system is fraught with issues including publication bias, inadequate reporting and lack of reproducibility.^7 8^ Implementing open science could reduce unnecessary duplication of research, thus saving time and money. Further, open science enhances transparency by making the various components of the research life cycle accessible thereby reducing bias but also driving innovation as others can use and adapt study data and materials. Open science also helps to support equity by reducing barriers in access to information. In medicine, this means that researchers and the public alike do not face barriers in accessing health information. Despite the growing impetus to implement open science globally, no country or discipline has achieved widespread adoption. There are several real and perceived challenges transitioning from our current norm of ‘closed research’. Issues including how to effectively create behaviour change to promote open science activities, how to train researchers on the formal practices involved with open science and how to reconcile openness with intellectual property, have all been raised as potential challenges.

The current study investigates cardiology researcher’s perceptions and practices related to open science. A recent cross-sectional study examining 232 publications in cardiology journals found that 96.6% (N=224/232) of papers audited did not have publicly available data, while 229/232 (98.7%) did not provide their analysis scripts, and 98.3% (228/232) did not refer to an accessible study protocol.^9^ Other related research has shown that study design and reporting elements to reduce bias in preclinical cardiology studies (eg, blinding, randomisation) are not common within the field and that this issue has persisted without much improvement year on year.^10^ Collectively, this work suggests that open science practices and related reporting and design best practices are not normative within cardiology. Given this reality, it is little wonder why concerns about reproducibility in the field persist.^11–13^ We know of no study to date that has surveyed cardiology researchers’ perceptions of open science. This is regrettable as knowledge of researchers’ perceptions of open science, and of barriers and facilitators to achieving openness, is essential to understand how to support the community to implement open science more fully. Other disciplines including social science,^14^ economics^15^ and psychology^16–18^ have conducted large-scale surveys of their researchers to determine the state of open science in their community. Such surveys can be used as a starting point to develop interventions to implement open science more effectively, but they can also serve to monitor open science over time with additional surveys that can be compared longitudinally. We used a cross-sectional online survey, sent to a randomly selected sample of corresponding authors of recent publications in well-known cardiology journals, to measure perceptions and practices related to open science. The study is descriptive, and we have no hypotheses. The survey is the first in a programme of research we are leading to target implementation of open science in cardiology.^19^

## Methods

### Transparency and ethics approval statement

This study received ethical approval from the Ottawa Health Science Network Research Ethics Board, Ottawa, Canada (20230437–01H). All study materials and data are available on the Open Science Framework^20^ along with the study registration: https://osf.io/v42u8/.^21^

### Study design

We conducted a cross-sectional online survey sent to a randomly selected sample of corresponding authors of publications in cardiology journals.

### Sampling framework

We identified the top 100 ‘Cardiology and Cardiovascular Medicine’ subject category journals from the SCImago journal ranking platform. This is a publicly available portal that draws from Scopus. We then extracted the corresponding author’s name and email from all articles published in these journals between 1 March 2021 and 1 March 2022. We included authors of all article types. For full details on our approach to extracting author emails please see online supplemental appendix 1. This is a convenience sample, because the work is descriptive and we are not conducting any inferential tests, we did not conduct a power analysis.

10.1136/openhrt-2023-002433.supp1 Supplementary data

### Participant recruitment

This closed survey was sent to researchers who we identified through our sampling framework. Potential participants received an email including an approved recruitment script that explained the study’s aim and invited them to complete our anonymous online survey. Involvement in the survey served as implied consent. There was no incentive to take part in the survey.

We used Mail Merge software to send emails to the authors in our sample. We sent three reminder emails to participants at weekly intervals from the original invitation to encourage responses and closed the survey 4 weeks after the initial invitation was received. After de-duplication of repeated emails, we sent our recruitment script to a total of 9594 researchers. We received 844 bounce backs, meaning a total our sample was 8750 researchers.

### Survey

The full survey is available in online supplemental appendix 2. Participants were asked six demographic questions (eg, gender, age). Following this, they responded to three questions about their research expertise and role. Then, participants were asked to indicate their familiarity with open science. Subsequent questions asked about participants’ training related to open science. Participants were presented with definitions of open access publishing, preprints, data sharing, materials sharing, protocol registration, reporting guidelines and patient engagement, and asked whether they had experience performing the practice and what barriers they face to so. Most of the questions were multiple choice and participants could navigate through a back button. Prior to completion, the survey was pilot tested by two cardiology researchers for clarity and format, with their feedback integrated into the design. We estimate that completing the survey took 10 min. Participants had the option of skipping any questions that they did not wish to answer.

10.1136/openhrt-2023-002433.supp2 Supplementary data

### Data analysis

Data analysis was conducted using Excel. We report basic descriptive statistics (eg, counts, percentages). Rather than conducting χ^2^ Crosstabs tests to test for group differences in responses (eg, considering gender, career stage, cardiology subdiscipline) as per our protocol, we have provided descriptive tables of these group differences given modest group sizes. For text-based responses, two members of the research team conducted a thematic content analysis. To do so, each researcher coded responses separately. Following a discussion and iterative updates to obtain a consensus on the codes, they were conceptually organised into topic areas and defined and explained in tables for reporting.

## Results

### Demographics

A total of 198 individuals completed the survey (response rate 2.3%). Participants tended to be men (N=153, 77%) and based in North America (N=97, 49%). Most participants reported to be faculty members/primary investigators (N=152, 77%), primarily working in clinical research (N=127, 64%) and that cardiovascular research was their main research area (N=178, 90%). For complete demographics, please see table 1.

**Table 1:** Participant demographics

Item	Response options	N	%
Which describes you best?	Male	153	77
	Female	43	22
	Non-binary	1	1
	Blank	1	1
What is your age group?	18–24	1	1
	25–34	24	12
	35–44	65	33
	45–54	50	25
	55–64	33	17
	65 or older	24	12
	Blank	1	1
Do you self-identify as having a disability?	Yes	3	2
	No	192	97
	Prefer not to say	2	1
	Blank	1	1
Do you identify as being part of a visible minority group?	Yes	25	13
	No	165	83
	Prefer not to say	4	2
	Blank	4	2
Do you currently identify as a caregiver (ie, parenting kids under 18, caring for elderly relatives)?	Yes	95	48
	No	98	49
	Prefer not to say	1	1
	Blank	4	2
Where are you located?	North America	97	49
	Asia	11	6
	South America	7	4
	Europe	72	36
	Australasia	9	5
	Africa	1	1
	Blank	1	1
Which of the following describes you best?	Faculty member/primary investigator	152	77
	Postdoctoral fellow	13	7
	Graduate student	9	5
	Scientist in third sector (eg, non-governmental organisation, non-profit)	2	1
	Research support staff (eg, research manager, research associate, technician)	6	3
	Scientist in industry	3	2
	Government scientist	6	3
	Other	6	3
	Blank	1	1
Which of the following best describes your primary research area?	Clinical research	127	64
	Health systems research	13	7
	Epidemiological research	15	8
	Preclinical research – in vivo	23	12
	Methods research	6	3
	Preclinical research – in vitro	6	3
	Other	7	4
	Blank	1	1
Is cardiovascular research your main area of research?	Yes	178	90
	No	20	10

### Open science familiarity, training and incentivisation

Participants had a mean response of 6.8 (N=197, SD=1.8) on a 9-point scale with endpoints, not at all familiar (1) and extremely familiar(9), when indicating how familiar they were with open science. When asked about where they obtained open science training most participants indicated this was done on the job self-initiated while conducting research (n=103, 52%), or that they had no formal training with respect to open science (n=72, 36%). Participants indicated their top format preference for training related to open science would be a website of resources. Additional funding to perform open science practices was the top incentive listed by participants to encourage them to apply more open science practices (N=154, 78%). More than half of participants indicated they would benefit from practical support from their institution on how to perform open science practices (N=106, 54%). Funders and research institutions were the top indicated stakeholders in terms of which has the most ability to create policies that result in successful uptake of open science. For complete results please see table 2.

**Table 2:** Open science education and motivation

Item	Responses	Yes (N)	%
Most of my training with respect to open science has been learnt:	On the job self-initiated while conducting research	103	52
	I have no formal training with respect to open science	72	36
	Through mentorship directly from my supervisor and/or peers	11	6
	Via formal coursework/workshops instructing about open science	8	4
	Other	3	2
	Blank	1	1
**Item**	**Rank**	**Responses**	
If you were to engage in training related to open science, which format of training would be your preference? (top 3)	1	A website of resources	
	2	An online webinar/recording	
	3	A short online course of six sessions (asynchronous)	
**Item**	**Responses**	**Yes (N)**	**%**
Which of the following incentives would result in you applying more open science practices?	Clearer communication about why open science is valuable for research	77	39
	Practical support from my institution to conduct open science	106	54
	Additional funding to perform open science practices	154	78
	Additional training on how to perform open science practices	61	31
	Having a staff trained on open science practices	53	27
	A way to get recognised for my performance of open science practices when I am being hired/promoted/tenured]	58	29
	Other	9	5
**Item**	**Rank**	**Responses**	
Rank order the stakeholders below in terms of which you feel has the most significant impact on creating policies that result in successful uptake of open science.	1	Funders	
	2	Research institutions	
	3	Scholarly journals	

Free text responses to the item asking about the best ways to promote open science were coded into 25 unique codes. These codes were then thematically grouped which resulted in seven categories: (1) Finances, (2) Incentives, (3) Policy and guidance, (4) Support, (5) Culture change, (6) Perceived concerns and (7) Other. Illustrative examples of each theme are provided in table 3.

**Table 3:** Thematic analysis of best ways to promote open science

Theme	Code	N (137)	%	Example
Finances	Provide funding	30	21.9	‘Provide funding at the institutional level to support it’
	Make it affordable	11	8.0	‘make it much less expensive’
	Do not charge fees to researchers	7	5.1	‘Don't charge scientists to do it’
Incentives and rewards	Incentivise open science practices	4	2.9	‘The incentive has to be correct, but one can not expect to give data away (as some initiatives almost look like).’
	Use novel metrics	4	2.9	‘Use novel metrics (Altmetrics) instead of impact factors.’
	Support broader types of publication models	3	2.2	‘Creation of open repository for scientific publications, and promotion of the repository through media.’
	Show value to researchers	6	4.4	‘Have research funders and universities promote it more, explain what it entails, and the benefits to the researcher and science.’
	Provide recognition for open science activities	3	2.2	‘Ensure that those who have spent years dedicated to generating the data continue to receive primary academic/ institutional credit for the work.’
Policy and process changes	Create mandates	11	8.0	‘Embed it as a requirement in nationally funded research’
	Improve peer review	2	1.5	‘Make it peer-reviewed.’
	Disseminate guidelines	2	1.5	‘Emphasize the importance of open science in society guidelines.’
Support	Facilitate the process	7	5.1	‘Institutional arrangements with publishers to ensure free Open Access publication in hybrid form’
	Provide training	12	8.8	‘undergraduate training’
	Knowledge sharing of practices	2	1.5	‘give example on how this will give access to data and protocols develop elsewhere.’
Culture change	Change journal culture	8	5.8	‘Change the journal culture.’
	Consider geographic realities	2	1.5	‘researchers from LMICS would simply publish under a subscription model.’
	Research culture change	4	2.9	‘Making data available would be the single step. However, the converse is that data is a scientists 'currency' for advancing their own research and exposes one to having ideas stolen.’
Perceived concerns	Address low quality journals	4	2.9	‘Open science is hampered by all the really bad journals promoting open-access paid articles.’
	Alleviate concern of misinformation	3	2.2	‘Alleviate concerns that data will not be misinterpreted, and reports will not have incorrect conclusions’
	Ensure open science is rigorous	3	2.2	‘I am not an unconditional fan of opens science as it is being promoted presently and therefore do not think it is appropriate to promote it without further consideration as it is currently done’
Other	Other	9	6.6	‘The biggest barrier, by far, is the risk associated with open data from institutional review boards and the privacy act.’

### Open science performance

Most participants reported having experience publishing an article open access (N=168, 85%) and using a reporting guideline (N=123, 62%). Roughly half of researchers reported that they had experience registering a study protocol (N=106, 54%) or engaging patients or members of the public in research (N=96, 48%). Fewer researchers reported experience sharing study materials (n=54, 27%), making a preprint (N=49, 25%) or sharing study data (N=48, 24%). Please see table 4.

**Table 4:** Open science performance

Item	Responses	N	%
In that past 12 months have you published an article ‘open access’?	Yes	168	85
	No	28	14
	I do not know	1	1
	I have not published a research paper in the past 12 months	1	1
In the past 12 months have you made a preprint prior to publishing an article?	Yes	49	25
	No	139	70
	I do not know	7	4
	Blank	3	2
In that past 12 months have you shared the raw data (all data necessary for reproducing the research) underpinning a study at the time of publication?	Yes	48	24
	No	147	74
	I do not know	1	1
	Blank	2	1
In the past 12 months have you shared the study materials underpinning a study at the time of publication?	Yes	54	27
	No	131	66
	I do not know	9	5
	Blank	4	2
In that past 12 months have you registered a study protocol for any research project you are working on?	Yes	106	54
	No	82	41
	I do not know	1	1
	I have not initiated a research study in the past 12 months	8	4
	Blank	1	1
In that past 12 months have you explicitly used and referenced a reporting guideline checklist in any research report you have published?	Yes	123	62
	No	66	33
	I do not know	6	3
	I have not published a research paper in the past 12 months	1	1
	Blank	2	1
In that past 12 months have you engaged patients or members of the public in any research you have conducted?	Yes	96	48
	No	94	47
	I do not know	3	2
	I have not conducted a research project in the past 12 months	3	2
	Blank	2	1

### Barriers to open science

A diversity of barriers to each of the open science practices presented to participants were acknowledged. For the complete results, please see table 5. When we asked participants about barriers to publishing their work open access, the top barrier identified was funding to support open access article processing charges (N=149, 75%). One-fifth of participants also indicated that they did not perceive their institution valued open access publishing (N=40, 20%). When asked about the barriers to creating a preprint almost half of participants indicated that they felt there were potential harms associated with work that has not been peer reviewed (N=91, 46%). Other key barriers included that participants worried that making a preprint would reduce their chances of the work being accepted at a peer reviewed journal (N=73, 37%), and that they did not see the benefit of making a preprint (N=71, 36%).

**Table 5:** Barriers to open science

Barrier	N	%
Open access publishing		
The journals in my area do not use an open access publishing model	15	8
I do not know how to self-archive a paper to make it open access	24	12
I do not see the benefit of making an article open access	15	8
I do not think my institution values me doing this	40	20
I do not have funding to support the article processing charges that are common at open access journals	149	75
I do not perceive any of the above as issues to publish open access	20	10
Other	21	11
Preprints		
I do not really know how to make a preprint	52	26
I do not have time to make preprints	35	18
I worry making a preprint will reduce my chances of the work being accepted at a peer reviewed journal	73	37
I do not see the benefit in making a preprint	71	36
I do not think my institution values me making a preprint	55	28
I think there are potential harms associated with sharing work that has not been peer reviewed	91	46
My institution has an internal process for posting preprints that makes the process very time consuming	7	4
Other	13	7
Data sharing		
I do not know how to prepare my data appropriately for sharing	45	23
I do not have time to prepare my data for sharing	59	30
I do not know where to share my data	44	22
I do not feel I will get recognition for sharing my data	65	33
My institutional ethics board will not allow me to share my data	58	29
My research consent form specifies I will not share the data	48	24
I am concerned about patient privacy if I share my data	72	36
Concerns about intellectual property control	94	47
Concerns about being scooped	56	28
Concerns about unintended use of secondary data	88	44
Concerns about misinterpretation of the data	75	38
Concerns others may discover errors in the data	15	8
Other	23	12
Materials sharing		
I do not know how to prepare my study materials for sharing	38	19
I do not have time to prepare my study materials for sharing	43	22
I do not know where to share my study materials	46	23
I do not think there is value for others in me sharing my study materials	21	11
There is no appropriate infrastructure available for me to share my study materials	49	25
The costs to share my study materials are a barrier	41	21
I do not feel I will get recognition for sharing my study materials	51	26
My institutional ethics board will not allow me to share my study materials	26	13
I am concerned about patient privacy if I share my study materials	35	18
Concerns about intellectual property control	63	32
Concerns about being scooped	35	18
Concerns about unintended use of materials	54	27
Concerns about misinterpretation of the materials	40	20
There are not any trained staff to guide me about it	21	11
Other	20	10
**Protocol registration**		
I do not know how to create a study registration	13	7
I do not know what platform to use to register my study	24	12
I do not have time to register my studies	33	17
I do not feel I will get recognition for taking the time to register my studies	24	12
I do not think that my institution prioritises study registration	24	12
I worry that I will be scooped if I share my study plan before publishing results	22	11
I do not think there is value for others in me registering my studies	18	9
I do not think my research area lends itself well to registering protocols	20	10
Other	23	12
Reporting guidelines		
I do not know where to find the relevant reporting guideline	23	12
I do not know how to use reporting guidelines	17	9
I do not have time to use reporting guidelines	23	12
I do not see the value in using reporting guidelines	21	11
I do not feel I will get recognition for taking the time to user reporting guidelines	27	14
I do not think my institution prioritises the use of reporting guidelines	17	9
Other	35	18
Patient and public involvement		
I do not know how to identify patients/public members to contribute	33	17
I do not know how to incorporate patients/public members in my research	48	24
I do not have time to incorporate patients/public members in my research	30	15
I do not see the value in incorporating patients/public members in my research	20	10
I do not feel I will get recognition for taking the time to incorporate patients/public members in my research	28	14
I do not think my institution prioritises incorporating patients/public members in my research	23	12
Other	35	18

When asked about barriers to sharing study data openly almost half of participants indicated they had concerns about intellectual property control (N=94, 47%). Other key barriers were participants’ concern about unintended use of secondary data (N=88, 44%) and concerns about misinterpretation of the data (N=75, 38%). Participants also raised concerns about intellectual property (N=63, 32%) and concerns about unintended use (N=54, 27%) as key barriers to materials sharing.

When asked about barriers to protocol registration, use of reporting guidelines and patient and public involvement, no overwhelming majority emerged for a particular item. The top barriers noted were that participants do not have time to register studies (N=33, 17%), participants do not feel they get recognition for taking time to use reporting guidelines (N=27, 14%) and that participants do not know how to incorporate patients/public members in their research (N=48, 24%).

## Discussion

We report the results of a survey of the cardiovascular community’s perceptions and experiences with open science. Our results compliment previous discipline specific efforts in social science,^14^ economics^15^ and psychology^16–18^ which have begun to provide data about the unique challenges of implementing and fostering open science in particular disciplines. Given the prevalence of cardiovascular diseases globally efforts to embed open science within this discipline have great potential to increase the useability and integrity of research in this area and ultimately to have a positive downstream impact on patient treatment and prevention of cardiovascular disease. Our findings provide an important baseline that can be used to track progress in open science implementation over time.

We found that most participants had either no formal open science training or had obtained training on the job on their own. This suggests that most researchers in the cardiovascular research community are figuring out open science as they encounter it, rather than any sort of cohesive community approach to implementation. This is especially concerning since participants also indicated that clearer communication about why open science is valuable for research would incentivise them to implement open science. Together, it suggests that participants need to be better and more systematically supported.

Participants indicated funding was the most essential incentive to adopt open science. The need for funding was also reflected in the thematic analysis of what is needed to best promote open science, where three codes related to financing open science. While much of this discussion focused on practicing open access publishing, which typically is associated with an article processing charge, the call for funding to hire personnel to carry out open science activities (eg, data management) was also made. The second most important incentive among participants was support from their institutions to conduct open science. Such support may take the form of toolkits or training, but support in the form of personal was again noted as valuable.

Participants indicated that they perceived funders had the most significant impact on creating policies that result in successful uptake of open science. This suggests that the community feels the need to respond to funder policies; however, when we examine the rates of self-reported performance of open science practices that are commonly mandated, we see a gap in performance. Overall, rates of self-reported performance of open science practices were limited. Eighty five per cent of respondents indicated they had published an open access article in the past year, while 62% indicated they had used a reporting guideline checklist. Mandates for both of these practices at the funder and journal level, respectively, are the norm.^22–24^ Funders implementing audit of the open science practices they mandate may help to ensure these and other practices are being implemented optimally.

About half of participants additionally indicated that they had registered a study protocol. This is interesting, given that mandates only exist for clinical trials,^25 26^ suggesting that the broader recognition of publication bias and selective outcome reporting^27 28^ may be leading to more general study registration although still at suboptimal levels. Almost half of participants indicated that they had engaged patients in research, yet 24% said they did not know how to incorporate patients/public members suggesting that there remains a need for awareness raising. Rates of the remaining open science practices were comparatively low, suggesting that they are even less embedded into the ethos of the average cardiovascular researcher.

When considering barriers to implementing each of the various open science practices, participants noted concerns that represent a lack of understanding or expertise on open science topics. For example, 37% worried that making a preprint would harm their chances of later publishing, despite the fact that preprints are nearly uniformly accepted at biomedical journals and that there are tools to check this.^29^ Another example is the concern about intellectual property control when sharing data and materials, which may suggest a need to outreach on how open science is compatible with a pathway for collaborative R&D.^30^

As a next step the barriers common to participants for each of the open science practices examined can be used to develop interventions to improve performance on the practice. Our survey will serve as a baseline for the community to track its progress on implementing open science but also on tracking what barriers persist and change over time to be responsive to the needs of the community. Furthermore, the cardiovascular research community might accelerate the creation and implementation of an international on open science strategy. A cardiovascular community open science strategy created jointly by funders, journals, scholarly, societies, patient diseases communities and related stakeholders could help to ensure that open science policy and practice was prioritised and that actions taken to drive improvements could be shared to reduce duplication of effort and streamline behaviour change.

While our study benefited from a broad sampling strategy and diverse participation, it has several limitations. Given the increasing mandates for open science practices it is possible that some participants did not feel comfortable providing answers that presented themselves in an unfavourable way. Survey question answer responses, particularly those presented when asking about barriers to the various open science practices, may not have fully represented participants’ views and may potentially have been interpreted differently by different participants. Finally, while the random sampling strategy we undertook allowed us to sample a diverse range of cardiovascular researchers, it is possible that those that responded to our survey, which was not incentivised in anyway, may differ in some way from those who opted not to respond. This selection bias limitation is a common weakness of survey designs, and we have no way of knowing if our sample matched the population of potential participants we invited to complete the survey. The response rate was modest and we cannot be certain that the data is a representative sample of the global cardiovascular community. The fact that we administered our survey in English will have reduced participation and biased our sample towards certain jurisdictions. The shared study materials, including surveys which can be translated and/or adapted, can be used in future research to sample more equitably.

We hope these findings will provide valuable data to discuss as a cardiovascular research community and as we endeavour to bring the community together to contribute to a roadmap to implanting open science.^19^

## Data Availability

Data are available in a public, open access repository. Data is openly available here: https://osf.io/v42u8/.
