# Supplementary material for: Cardiology researchers’ practices and perceived barriers to open science: an international survey
Source: Open Heart. 2024 Jan 17;11(1):e002433. doi: 10.1136/openhrt-2023-002433 (PMC10806507; doi:10.1136/openhrt-2023-002433)
Supplement: Supplementary data [file openhrt-2023-002433supp001.pdf]

Appendix 1

Journal Search

We will obtain a list the top 100 ranked "Cardiology and Cardiovascular Medicine" subject category journals from the SCImago journal ranking platform.

Article Retrieval

We will search for all articles published in each journal using the search strategy *1234567.jc.* where "1234567" is the NLM ID of the journal. Where NLM ID is not available, we will use the syntax "Name of journal".nj.

We will run search for each journal separately. After each search, we will sort the results by Entry date (descending) and export all publications between March 2021-March 2022.

Email Retrieval

All retrieved articles will be re-imported to Zotero to retrieve PMID numbers. The list of PMID numbers will be exported as an .csv file and input into an R script (built based on the easyPubMed package) to retrieve the authors' name, affiliation institutions and email addresses. We will then de-duplicate emails.
