# Supplementary material for: Cardiology researchers’ practices and perceived barriers to open science: an international survey
Source: Open Heart. 2024 Jan 17;11(1):e002433. doi: 10.1136/openhrt-2023-002433 (PMC10806507; doi:10.1136/openhrt-2023-002433)
Supplement: Supplementary data [file openhrt-2023-002433supp002.pdf]

Appendix 2: Study survey

Demographics

1. Which describes you best?
    - Female
    - Male
    - Non-binary
    - Prefer to self-describe ______
    - Prefer not to say
    -
2. What is your age group?
    - 18-24
    - 25-34
    - 35-44
    - 45-54
    - 55-64
    - 65 or older
    - Prefer not to say

3. Do you identify as being part of a visible minority group?
    - Yes
    - No
    - Prefer not to say

4. Do you self-identify as having a disability?
    - Yes
    - No
    - Prefer not to say

5. Do you currently identify as a caregiver (i.e., parenting kids under 18, caring for elderly relatives)?
    - Yes
    - No
    - Prefer not to say

6. Where are you located?

- Africa
- Asia
- Australasia
- Europe

- North America
- South America

Research demographics

7. Which of the following describes you best?
- Graduate student
- Postdoctoral fellow
- Faculty member/PI
- Research support staff (E.g., research manager, research associate, technician)
- Scientist in industry
- Scientist in third sector (E.g., NGO, non-profit)
- Government scientist
- Other, please specify

8. Which of the following best describes your primary research area?
- Clinical research
- Preclinical research – in vivo
- Preclinical research – in vitro
- Health systems research
- Methods research
- Epidemiological research
- Other, please specify

9. Is cardiovascular research your main area of research?
   Yes
   No

Open Science practice and experience

"Open science is an umbrella term that reflects the idea that scientific knowledge of all kinds, where appropriate, should be openly accessible, transparent, rigorous, reproducible, replicable, accumulative and inclusive, all which are considered fundamental features of the scientific endeavour. Open science consists of principles and behaviours that promote transparent, credible, reproducible and accessible science. Open science has six major aspects: open data, open methodology, open source, open access, open peer review and open educational resources." (Source: FORRT Glossary)

1. How familiar are you with the concept of open science overall? (1-9-point scale; Not at all familiar – Moderately familiar – Extremely familiar)
2. *Note: There was an item in the protocol additional to the general item above asking participants to respond to how familiar they are with each of the below open science practices, but due to a technical glitch in survey development, it was not presented to all participants*

Item	Definition
Open access	"Free availability of scholarship on the public internet, permitting any users to read, download, copy, distribute, print, search, or link to the full texts of these research articles, crawl them for indexing, pass them as data to software, or use them for any other lawful purpose, without financial, legal, or technical barriers other than those inseparable from gaining access to the internet itself" (Boai, 2002).
Preprints	A publicly available version of any type of scientific manuscript/research output preceding formal publication, considered a form of Green Open Access. Preprints are usually hosted on a repository (e.g. arXiv) that facilitates dissemination by sharing research results more quickly than through traditional publication. Preprint repositories typically provide persistent identifiers (e.g. DOIs) to preprints. Preprints can be published at any point during the research cycle, but are most commonly published upon submission (i.e., before peer-review). Accepted and peer-reviewed versions of articles are also often uploaded to preprint servers, and are called postprints.
Open data	Open data refers to data that is freely available and readily accessible for use by others without restriction, "Open data and content can be freely used, modified, and shared by anyone for any purpose" (https://opendefinition.org/). Open data are subject to the requirement to attribute and share alike, thus it is important to consider appropriate Open Licenses. Sensitive or time-sensitive datasets can be embargoed or shared with more selective access options to ensure data integrity is upheld.
Open materials	Author's public sharing of materials that were used in a study, "such as survey items, stimulus materials, and experiment programs" (Kidwell et al., 2016, p. 3). Digitally-shareable materials are posted on open access repositories, which makes them publicly available and accessible. Depending on licensing, the material can be reused by other authors for their own studies. Components that are not digitally-shareable (e.g. biological materials, equipment) must be described in sufficient detail to allow reproducibility.
Registration	The practice of publishing the plan for a study, including research questions/hypotheses, research design, data analysis before the data has been collected or examined. It is also possible to preregister secondary data analyses (Merten & Krypotos, 2019). A preregistration document is time-stamped and typically registered

	with an independent party (e.g., a repository) so that it can be publicly shared with others (possibly after an embargo period). Preregistration provides a transparent documentation of what was planned at a certain time point, and allows third parties to assess what changes may have occurred afterwards. The more detailed a preregistration is, the better third parties can assess these changes and with that the validity of the performed analyses. Preregistration aims to clearly distinguish confirmatory from exploratory research.
Reporting guidelines	A reporting guideline is a “checklist, flow diagram, or structured text to guide authors in reporting a specific type of research, developed using explicit methodology.” (EQUATOR Network, n.d.). Reporting guidelines provide the minimum guidance required to ensure that research findings can be appropriately interpreted, appraised, synthesized and replicated. Their use often differs per scientific journal or publisher.
Patient and public involvement	Active research collaboration with the population of interest, as opposed to conducting research “about” them. Researchers can incorporate the lived experience and expertise of patients and the public at all stages of the research process. For example, patients can help to develop a set of research questions, review the suitability of a study design, approve plain English summaries for grant/ethics applications and dissemination, collect and analyse data, and assist with writing up a project for publication. This is becoming highly recommended and even required by funders (Boivin et al., 2018).

3. Most of my training with respect to open science has been learned:
    a. On the job self-initiated while conducting research
    b. Via formal coursework/workshops instructing about open science
    c. Through mentorship directly from my supervisor and/or peers
    d. Other, please specify:
    e. I have no formal training with respect to open science

4. If you were to engage in training related to open science, which format of training would be your preference? (Rank order)

    a. A website of resources
    b. A handbook of resources
    c. A short online course of six sessions (asynchronous)
    d. A short online course of six sessions (live)
    e. An online webinar/recording

    f. An in-person lecture at my institution
    g. An in-person workshop at my institution

5. Which of the following incentives would result in you applying more open science practices? (Check all that apply)
    a. Clearer communication about why open science is valuable for research
    b. Practical support from my institution to conduct open science (E.g., a person I could turn to ask questions about the practicalities of performing open science)
    c. Additional funding to perform open science practices (E.g., funding for open access charges, funding for staff to help prepare data for open sharing)
    d. Additional training on how to perform open science practices
    e. Having a staff trained on open science practices
    f. A way to get recognized for my performance of open science practices when I am being hired/promoted/tenured
    g. Other, please specify

6. Rank order the stakeholders below in terms of which you feel has the most significant impact on creating policies that result in successful uptake of open science.
    a. Research institutions
    b. Funders
    c. Scholarly journals
    d. Scholarly societies

7. What do you think is the best way to promote open science? (Free text)

Open access

8. In that past 12 months have you published an article ‘open access’?
    a. Yes
    b. No
    c. I have not published a research paper in the past 12 months
    d. I don’t know

9. Which of the following are barriers for you with respect to publishing open access? (Check all that apply)

    a. The journals in my area don’t use an open access publishing model
    b. I don’t know how to self-archive a paper to make it open access
    c. I don’t see the benefit of making an article open access
    d. I don’t think my institution values me doing this

e. I don’t have funding to support the article processing charges that are common at open access journals
f. I do not perceive any of the above as issues to publish open access
g. Other, please specify

Preprints

10. In the past 12 months have you made a preprint prior to publishing an article?
    a. Yes
    b. No
    c. I have not published a research paper in the past 12 months
    d. I don’t know

11. Which of the following are barriers for you with respect to creating preprints? (Check all that apply)
    a. I don’t really know how to make a preprint
    b. I don’t have time to make preprints
    c. I worry making a preprint will reduce my chances of the work being accepted at a peer reviewed journal
    d. I don’t see the benefit in making a preprint
    e. I don’t think my institution values me making a preprint
    f. I think there are potential harms associated with sharing work that has not been peer reviewed
    g. My institution has an internal process for posting preprints that makes the process very time consuming
    h. Other, please specify

Open Data

12. In that past 12 months have you shared the raw data (all data necessary for reproducing the research) underpinning a study at the time of publication?
    a. Yes
    b. No
    c. I have not published a research paper in the past 12 months
    d. I don’t know

13. Which of the following are barriers for you with respect to you sharing the raw data from your research when publishing? (Check all that apply)

a. I don’t know how to prepare my data appropriately for sharing
b. I don’t have time to prepare my data for sharing

c. I don't know where to share my data
d. I don't feel I will get recognition for sharing my data
e. My institutional ethics board will not allow me to share my data
f. My research consent form specifies I will not share the data
g. I am concerned about patient privacy if I share my data
h. Concerns about intellectual property control
i. Concerns about being scooped
j. Concerns about unintended use of secondary data
k. Concerns about misinterpretation of the data
l. Concerns others may discover errors in the data
m. Other, please specify

Open Materials

14. In that past 12 months have you shared the study materials underpinning a study at the time of publication?
    a. Yes
    b. No
    c. I have not published a research paper in the past 12 months
    d. I don't know

15. Which of the following are barriers for you with respect to you sharing study materials from your research when publishing? (Check all that apply)

a. I don't know how to prepare my study materials for sharing
b. I don't have time to prepare my study materials for sharing
c. I don't know where to share my study materials
d. I don't think there is value for others in me sharing my study materials
e. There is no appropriate infrastructure available for me to share my study materials
f. The costs to share my study materials are a barrier
g. I don't feel I will get recognition for sharing my study materials
h. My institutional ethics board will not allow me to share my study materials
i. I am concerned about patient privacy if I share my study materials
j. Concerns about intellectual property control
k. Concerns about being scooped
l. Concerns about unintended use of materials
m. Concerns about misinterpretation of the materials
n. There aren't any trained staff to guide me about it
o. Other, please specify

Registration

16. In that past 12 months have you registered a study protocol for any research project you are working on?
    Yes
    No
    I have not initiated a research study in the past 12 months
    I don't know

17. Which of the following are barriers for you with respect to registering your study protocol prior to starting the research project? (Check all that apply)
    a. I don't know how to create a study registration
    b. I don't know what platform to use to register my study
    c. I don't have time to register my studies
    d. I don't feel I will get recognition for taking the time to register my studies
    e. I don't think that my institution prioritizes study registration
    f. I worry that I will be scooped if I share my study plan before publishing results
    g. I don't think there is value for others in me registering my studies
    h. I don't think my research area lends itself well to registering protocols
    i. Other, please specify

Reporting guidelines

18. In that past 12 months have you explicitly used and referenced a reporting guideline checklist in any research report you have published?
    Yes
    No
    I have not published a research paper in the past 12 months
    I don't know

19. Which of the following are barriers for you with respect to using reporting guidelines when reporting your research? (Check all that apply)
    a. I don't know where to find the relevant reporting guideline
    b. I don't know how to use reporting guidelines
    c. I don't have time to use reporting guidelines
    d. I don't see the value in using reporting guidelines
    e. I don't feel I will get recognition for taking the time to user reporting guidelines
    f. I don't think my institution prioritizes the use of reporting guidelines
    g. Other, please specify

Patient and public Involvement

20. In that past 12 months have you engaged patients or members of the public in any research you have conducted?

Yes
No
I have not conducted a research project in the past 12 months
I don't know

21. Which of the following are barriers for you with engaging patients or members of the public in your research? (Check all that apply)

    a. I don't know how to identify patients/public members to contribute
    b. I don't know how to incorporate patients/public members in my research
    c. I don't have time to incorporate patients/public members in my research
    d. I don't see the value in incorporating patients/public members in my research
    e. I don't feel I will get recognition for taking the time to incorporate patients/public members in my research
    f. I don't think my institution prioritizes incorporating patients/public members in my research
    g. Other, please specify

22. Is there anything else you want to share? (Free text)
